# LncRNA ZFPM2 silencing alleviates lung epithelial injury by inducing M2 macrophage polarization: possible implication in bronchopulmonary dysplasia

**DOI:** 10.3389/fped.2026.1921479

**Published:** 2026-10-06

**Authors:** Hanzhou Guan, Huaiqing Yin

**Affiliations:** 1 Department of Neonaology, Children’s Hospital Affiliated to Shanxi Medical University, Taiyuan, Shanxi, China; 2 Department of Pediatrics, First Hospital of Shanxi Medical University, Taiyuan, Shanxi, China

**Keywords:** bronchopulmonary dysplasia, high-throughput sequencing, inflammation, lncRNA ZFPM2, macrophage polarization

## Abstract

**Background:**

To screen the key long-chain non-coding RNA (LncRNA) closely related to the occurrence and development of Bronchopulmonary dysplasia (BPD), and explore its mechanism of affecting lung epithelial cell injury.

**Methods:**

Based on the BPD mouse model induced by lipopolysaccharide (LPS), LncRNA high-throughput sequencing was used to screen differentially expressed LncRNAs. GO and KEGG functional enrichment analysis were performed on differentially expressed target genes. The expressions of key LncRNA in LPS-induced MLE-12 and MH-S cells were verified. ZFPM2 specific siRNA (si-ZFPM2) and negative control (si-NC) plasmids were transfected into LPS-induced cells. Cells were divided into control group, LPS group, LPS + si-NC group, and LPS + si-ZFPM2 group. Cell vitality was measured by CCK-8 method, pro-inflammatory cytokines (IL-1β, TNF-α) and anti-inflammatory cytokines (IL-10, IL-4) were quantified, and M1 (iNOS, CD86) and M2 (CD206, Arg1) markers were detected. In MLE-12 cells, cell apoptosis rate was further detected, and the protein expressions of NLRP3 and caspase-1 were analysed. A Transwell co-culture system was established with MH-S cells in the upper chamber (treated as above) and MLE-12 cells in the lower chamber; the latter were harvested to assess viability, apoptosis, NLRP3/caspase-1 expression, and inflammatory cytokine levels.

**Results:**

A total of 1,290 differentially expressed LncRNAs and 4,084 differentially expressed mRNAs were screened by LncRNA sequencing. Functional enrichment analysis showed that these differential genes were closely related to the development of lung epithelial cells and macrophage polarization. The expression of ZFPM2 was markedly upregulated in BPD mouse lung tissue and in LPS-stimulated cells. In MH-S cells, silencing ZFPM2 significantly increased cell viability, decreased M1 markers (iNOS, CD86) and pro-inflammatory cytokines (TNF-α, IL-6, IL-1β), and increased M2 markers (CD206, Arg1) and anti-inflammatory cytokines (IL-4, IL-10). In MLE-12 cells, silencing ZFPM2 significantly improved viability, reduced apoptosis, and suppressed NLRP3/caspase-1 protein expression and IL-1β, IL-6, and TNF-α levels. In the co-culture system, silencing ZFPM2 significantly enhanced MLE-12 viability, reduced apoptosis and NLRP3/caspase-1 expression, lowered pro-inflammatory cytokines, and elevated anti-inflammatory cytokines.

**Conclusions:**

Silencing ZFPM2 can alleviate LPS-induced injury of pulmonary epithelial cells, which may be related to promoting the polarization of alveolar macrophages towards M2 type and inhibiting inflammatory response.

## 1 Introduction

Bronchopulmonary dysplasia (BPD) is the most common chronic lung disease in premature infants and an independent risk factor affecting their long-term cognitive development (1, 2). Simplified alveolar development is the core pathological feature of BPD, which is characterized by reduced number of alveoli, thickening of alveolar septa and impaired development of capillary network. These changes can lead to lifelong respiratory diseases such as asthma, decreased exercise ability, early-onset emphysema and pulmonary hypertension. The pathogenesis of BPD is complex, involving multiple factors such as oxygen poisoning, sepsis, and mechanical ventilation (3), among which inflammatory response is considered to be a key process mediating the pathological phenotype of BPD (4, 5). At present, there is no specific prevention or treatment plan for BPD, and the commonly used clinical treatment methods such as mechanical ventilation, dexamethasone and diuretics have limited improvement (6–8). Therefore, it is of great significance to deeply explore the pathological mechanism of BPD and explore new treatment strategies.

Long non-coding RNAs (lncRNAs) are a class of non-coding RNAs with a length exceeding 200 nucleotides, which are involved in the regulation of various biological processes. However, their role in the pathogenesis of BPD is not yet fully clear. Previous studies have shown that lncRNA-VSIG2-1:1 is upregulated in the lung tissues of BPD patients and in hypoxic-induced pulmonary microvascular endothelial cells (HPMECs), and the inhibition of lncRNA-VSIG2-1:1 promotes the proliferation, migration, and tube formation ability of HPMECs (9). LncRNA UC.375 is expressed at a lower level in the lung tissues of BPD mice, and silencing of uc.375 inhibits the proliferation of MLE-12 cells and promotes cell apoptosis (10). These studies suggest that lncRNAs may become potential targets for the treatment of BPD.

In this study, firstly, based on a BPD mouse model induced by lipopolysaccharide (LPS), differentially expressed lncRNAs were screened through lncRNA high-throughput sequencing and functional analysis. Furthermore, MLE-12 mouse lung epithelial cells and MH-S mouse alveolar macrophages were used to verify its mechanism *in vitro*, in order to provide a new target and theoretical basis for the diagnosis and treatment of BPD.

## 2 Methods

### 2.1 Grouping and intervention of animals

After 7 days of adaptive feeding (22–26℃, 50%～60%, 12:12 h light-dark cycle), specific pathogens (SPF) -grade C57BL/6 mice (8 weeks old, 4 females and 2 males) were caged and mated in a ratio of 2:1 to obtain newborn mice. Ten newborn mice were randomly divided into 2 groups (5 mice in each group): control group and BPD model group. The model group mice were intranasally instilled with lipopolysaccharide (LPS, 3 μg/10 μL) on day 3, 5, 7, and 10 after birth, while the control group mice were given the same volume of normal saline at the same time points. Lung tissues were collected on the 14th day after birth, blood was taken at the same time points and immediately processed, and then stored in liquid nitrogen. All animal experimental protocols were approved by the Animal Management Committee of Hubei Province and the Animal Ethics Committee of Wuhan Myhalic Biotechnology Co., Ltd. [Animal Use License Number: SYXK (E) 2023-0104; Ethical Approval Number: HLK-20231204-002]. During the experiment, the number of animals used and their suffering were minimized as much as possible. The animals were euthanized under deep anesthesia by dislocating the cervical vertebra.

### 2.2 HE staining

Take the right upper lobe lung tissue and fix it in 4% paraformaldehyde for 24 h. After gradient ethanol dehydration and paraffin embedding, prepare 4 μm thick continuous sections. The sections are stained with hematoxylin-eosin (H&E) for morphological observation of lung tissue and assessment of lung injury severity. All stained sections are observed and scored under an optical microscope by two researchers who are unaware of the grouping situation. According to the experimental guidelines for evaluating acute lung injury published by the American Thoracic Society (ATS), a semi-quantitative scoring system is used to evaluate the degree of lung injury (11, 12), with the evaluation indicators including: (1) neutrophil infiltration in the alveolar cavity; (2) neutrophil infiltration in the alveolar septum; (3) formation of hyaline membranes; (4) accumulation of protein debris/exudate in the alveolar cavity; (5) thickening of the alveolar septum (13). Each indicator is assigned a score of 0–4 (0: no lesion; 1: mild focal lesion; 2: moderate multifocal lesion; 3: severe diffuse lesion; 4: extremely severe diffuse lesion), and the total lung injury score is the sum of the scores of each indicator. The H&E staining and lung injury scoring results are used to verify the success of BPD model construction.

After model validation, the mice were euthanized by cervical dislocation, and the left lung tissue is rapidly placed in liquid nitrogen for rapid freezing, then transferred to a −80℃ refrigerator for subsequent LncRNA high-throughput sequencing analysis.

### 2.3 LncRNA high-throughput sequencing

Total RNA from lung tissues was extracted using TRIzol reagent (Invitrogen, USA) and the procedure was carried out strictly according to the instructions. The integrity and quality of the total RNA were evaluated using the Agilent 2,100 bioanalyzer (Agilent Technologies, USA). 1 μg of qualified total RNA was taken, rRNA was removed (some LncRNAs have a polyA tail similar to mRNA, removing rRNA can maximize the retention of LncRNAs with polyA tails), and the library was constructed using the TruSeq Stranded Total RNA Library Prep Gold (Illumina, 20020599) reagent kit. Subsequently, the sequencing library was prepared using the NEBNext® Ultra™ RNA Library Prep Kit for Illumina® (NEB, E7420, USA), and specific index sequences were added to each sample. The library fragments were purified using the AMPure XP system (Beckman Coulter, USA), and the double-stranded cDNA was digested with USER enzyme (NEB, USA) at 37 °C for 15 min, followed by 5 min of termination at 95 °C. Then, PCR amplification was performed. The PCR reaction was carried out using Phusion high-fidelity DNA polymerase, universal PCR primers, and index primers. The integrity of the library fragments and the size of the insert fragments were detected using the AATI analyzer, and the insert fragment size was preliminarily quantified using the Qubit 2.0 fluorescence meter and then diluted to 1.5 ng/*μ*L. The insert fragment size was then detected using the Agilent 2,100 bioanalyzer. After verification, the effective concentration of the library was accurately quantified using qRT-PCR (requiring >2 nM). Based on the effective concentration of the library and the target sequencing data volume, the samples were mixed, and dual-end 150 bp sequencing was performed on the Illumina NovaSeq 6,000 platform to obtain raw fastq format data.

The raw sequencing data were preprocessed using an internal Perl script. The reference genome and gene model annotation files were downloaded from the public genomic website, and the reference genome index was constructed and aligned using HISAT2 (v2.0.5). The read counts were calculated using StringTie (v1.3.3b) for each gene, and the expression was quantified using FPKM (the number of fragments per 1 million aligned reads per 1,000 base transcripts). The read counts were standardized using the edgeR (v3.22.5) software package (scaling normalization factor), and differentially expressed genes between the two groups were screened. The *P* values were corrected using the Benjamini-Hochberg method, and genes with *P* < 0.05 after correction were considered significantly differentially expressed mRNAs (DEmRNAs) and lncRNAs (DElncRNAs). The clusterProfiler (v3.8.1) software was used to perform GO (Gene Ontology), KEGG (Kyoto Encyclopedia of Genes and Genomes), and Reactome pathway enrichment analysis of the target genes of DEmRNAs and DElncRNAs, with a significance threshold of corrected *P* < 0.05.

### 2.4 qRT-PCR detection

Total RNA was extracted using the FastPure Cell/Tissue Total RNA Isolation Kit V2 (RC112-01, Vazyme) and resuspended in RNase-free H₂O. 5 μg of total RNA was taken and 3 μL of 5×gDNA digester Mix was added, followed by the addition of RNase-free H₂O to make a total of 15 μL. The mixture was shaken well and incubated at 42 °C for 2 min to remove residual genomic DNA. Then, 5 μL of 4×Hifair® Ⅲ SuperMix plus (11141ES60, YEASEN) was added. The reverse transcription was performed according to the following procedure: 25 °C for 5 min, 55 °C for 15 min, and 85 °C for 5 min. The PCR reaction system (20 μL) contained: 8 μL cDNA template, 10 μL Hieff® qPCR SYBR® Green Master Mix, 0.5 μL of each upstream and downstream primer, and RNase-free H₂O to make up to 20 μL. The reaction procedure was: 95 °C for 5 min of pre-denaturation; 95 °C for 5 s, 56 °C for 10 s, and 72 °C for 25 s for 40 cycles; finally, 65 °C for 5 s. The primer sequences are shown in Table 1. The relative expression level of the target gene was calculated using the 2^−*ΔΔ*Ct method, and normalized with GAPDH as the internal reference gene. Each sample was tested three times in duplicate.

**Table 1:** Sequences of qRT-PCR primers.

Gene	Sequence (5’ to 3’)	Length
Zfpm2	F: CCCGCCGTATCTGCTTAG	171
	R: GTCATAGACCCTTGCCCTTA	
iNOS	F: GCTAATGCGAAAGGTCA	122
	R: TGGTGTTGAAGGCGTAG	
CD86	F: ATGGGCTCGTATGATTGTTT	193
	R: TCTTCTTAGGTTTCGGGTGA	
CD206	F: GGTGGCTTATGGGATGTTT	108
	R: GGGTTCAGGAGTTGTTGTG	
Arg1	F: TTGGGAAGACAGCAGAGGA	107
	R: GTCAGTCCCTGGCTTATGG	
GAPDH	F: CCTTCCGTGTTCCTAC	152
	R: GACAACCTGGTCCTCA	

### 2.5 Construction of interference plasmids and cell transfection

Based on the ZFPM2 gene sequence (Transcript ID: ENSMUST00000229777.2), three interference targets and one negative control were designed and synthesized. They were constructed into the vector pLKO.1-EGFP-puro, resulting in the interference plasmid (si-ZFPM2) and the control plasmid (si-NC). 24 h before transfection, 5 × 10⁵ cells were inoculated into a 6-well plate containing 2 mL of complete medium. 4 μg of plasmid DNA was dissolved in 250 μL Opti-MEM, and 10 μL of Lipofectamine 2,000 was dissolved in 250 μL Opti-MEM, which were gently mixed separately and left at room temperature for 5 min. Then, the two were slowly mixed and left at room temperature for 20 min. 500 μL of the complex was added to the cell culture plate wells, gently shaken to mix well, and placed in a 37 °C, 5% CO₂ incubator. After 4 h of transfection, the fresh medium was replaced, and the cells were cultured for 72 h. The expression of the transferred gene was detected by qRT-PCR, and the best interference target was selected.

### 2.6 Cell grouping and treatment

#### 2.6.1 Cell grouping 1

MLE-12 lung epithelial cells (procell) were resuspended in 1 mL of culture medium, transferred to a culture flask, and 4 mL of complete culture medium was added. The cells were cultured in a 37 °C, 5% CO₂ incubator. After 24 h of stimulation with 10 μg/mL LPS (0000153963, Sigma), ZFPM2 expression was detected by qRT-PCR. The cells were divided into: control group (untreated), LPS group (10 μg/mL LPS stimulation for 24 h), LPS + si-NC group (transfection of empty plasmid followed by LPS stimulation for 24 h), and LPS + si-ZFPM2 group (transfection of si-ZFPM2 followed by LPS stimulation for 24 h).

#### 2.6.2 Cell grouping 2

MH-S alveolar macrophages cells (icell) were stimulated with 100 ng/mL LPS for 24 h. ZFPM2 expression was detected by qRT-PCR. The groups were the same as Cell grouping 1, with the LPS stimulation concentration being 100 ng/mL.

#### 2.6.3 Cell grouping 3

A Transwell co-culture system was established. MLE-12 cells were placed in the lower chamber (2 × 10⁴ cells, 700 μL), and MH-S cells were placed in the upper chamber (5 × 10^3^ cells, 200 μL). The cells in the upper chamber were treated according to Cell grouping 2, and then co-cultured in a 37 °C, 5% CO₂ incubator for 24 h. The cell culture plates were removed, the upper chamber was removed, the culture medium was discarded, 300 μL of CCK-8 solution was added to each well, and the cells were cultured for 4 h 100 μL was aspirated and measured at 450 nm on an enzyme-linked immunosorbent assay instrument.

### 2.7 CCK-8 detection

The cell viability was detected by the CCK-8 method. The logarithmic growth phase cells were digested and resuspended. They were inoculated into 96-well plates at an appropriate density (usually 1 × 10⁴ cells per well in 100 μL medium), with 3–5 replicate wells set for each group. The plates were cultured in a 37 °C, 5% CO₂ incubator until the corresponding time point. 10 μL of CCK-8 reagent was added to each well for detection. The mixture was gently shaken and incubated for 1–4 h. The absorbance (OD value) at 450 nm wavelength was measured using an enzyme detector, and corrected with 630 nm as the reference wavelength. The cell viability was calculated according to the following formula: Cell viability (%) = (Experimental group OD value—Blank group OD value)/(Control group OD value—Blank group OD value) × 100%.

### 2.8 Flow cytometry detection

The cell apoptosis was detected by the Annexin V-FITC/PI double staining method. The cells to be tested (including suspended and adherent cells) were collected, pre-cooled with PBS, washed, and resuspended in 1×Binding Buffer and adjusted to a density of approximately 1 × 10⁶ cells/mL. 100 μL of cell suspension (about 1 × 10⁵ cells) was taken, and 5 μL of Annexin V-FITC and 5 μL of PI staining solution were added. The mixture was gently mixed and incubated at room temperature in the dark for 15–20 min. 400 μL of 1×Binding Buffer was added to terminate the staining, and the sample was immediately analyzed on the instrument. The target cell population was circled in the FSC-H/SSC-H scatter plot to exclude cell debris and subcellular particles, and then the FSC-H/SSC-H scatter plot is used to exclude cell adhesions (doublets) to ensure that only a single intact cell is analyzed; In the FITC-H/PE-H scatter plot, the spontaneous fluorescence and single-stained samples of cells from the control group (Annexin V-FITC single-stained, PI single-stained) were used for fluorescence compensation adjustment and quadrant boundary setting: Annexin V⁻/PI⁻ were live cells (Q3), Annexin V⁺/PI⁻ were early apoptotic cells (Q4), Annexin V⁺/PI⁺ were late apoptotic cells (Q2), and Annexin V⁻/PI⁺ were necrotic cells (Q1). The total apoptosis rate was the sum of the early and late apoptosis rates (Q4 + Q2).

### 2.9 Western blot detection

The lung tissue or cell samples were treated with a lysis buffer containing protease and phosphatase inhibitors, and protein quantification was performed using the BCA method. A 10% SDS-PAGE gel was prepared and placed in an electrophoresis chamber, with the electrophoresis buffer added to each well. 20 μg of protein was added to each well, and electrophoresis was performed at 300 V for 30 min. The membrane was transferred using the wet transfer method and placed in the blocking solution at room temperature for 10 min to block. One antibody (caspase-1: PAB46174, 1:1000, Bioswamp; NLRP3: PAB37930, 1:1000, Bioswamp; *β*-Actin: 66009-1-lg, 1:10000, Proteintech) was incubated at room temperature for 1 h, washed with PBST three times for 5 min each time. The secondary antibody (SAB43714, 1:20000, Bioswamp) was added at room temperature for 1 h, washed three times with PBST for 5 min each time. The chemiluminescence was developed using ECL reagent, and detected by an automatic chemiluminescence analyzer. The gray value of the band was read by TANON GIS software.

### 2.10 ELISA detection

The content of TNF-α, IL-6 and IL-1β in the cell culture supernatant was detected using the double antibody sandwich ELISA method. The standard substances were diluted in a ratio to establish a concentration gradient. The enzyme-labeled plate was set up with standard substance wells, blank wells and sample wells. 50 μL of different concentrations of standard substances were added to the standard substance wells, and 40 μL of the sample was added to the sample wells, followed by 10 μL of biotin-labeled detection antibody. Except for the blank wells, 50 μL of enzyme-labeled reagent was added to each well, and the plate was sealed and incubated at 37 °C for 30 min. The liquid was discarded, and the plate was washed five times with the washing solution (each time for 30 s and then dried). 50 μL of each well was added with color developer A and B, and the reaction was allowed to proceed for 10 min in the dark. 50 μL of stop solution was added to terminate the reaction, and the OD value was measured at 450 nm wavelength. A quadratic regression equation was fitted based on the standard substance concentration and OD value, and the concentration of cytokines in the samples was calculated.

### 2.11 Statistical analysis

All experiments were independently repeated at least 3 times, and the results were expressed as the mean ± standard deviation (mean ± SD). Statistical analysis was performed using GraphPad Prism 9 and SPSS 22.0 software. Comparisons between two groups were carried out using the independent samples t-test. The comparisons among multiple groups were conducted using one-way analysis of variance, and the *post hoc* pairwise comparisons were performed using the Tukey HSD method for multiple comparison correction. Differences were considered statistically significant when *P* < 0.05, *P* < 0.01, or *P* < 0.001.

## 3 Results

### 3.1 Screening and identification of LncRNA ZFPM2

As shown in Figures 1A,B, a BPD mouse model was constructed by instilling LPS into the trachea of mice. The results revealed that the lung tissue of mice in the control group had intact bronchial structures and orderly arrangement of bronchial epithelial cells. Compared with the control group, some areas of the alveoli gradually expanded and even ruptured, forming cyst-like dilated alveoli with thickened alveolar walls and dilated alveolar capillaries in the model group, and there were also necrotic and shedding alveolar epithelial cells and a small amount of red blood cells in the alveolar cavities in some areas (Figure 1C). Compared with the control group, the lung tissue pathological score of the model group mice significantly increased (Figure 1D). To screen the key LncRNAs related to BPD onset, this study conducted transcriptome analysis of the lung tissue of BPD model mice through high-throughput sequencing. Compared with the control group, a total of 1,290 differentially expressed LncRNAs (|log2FC| > 1, *P* < 0.05) were identified, among which 696 were upregulated and 594 were downregulated (Figure 1E, volcano plot); 4,084 differentially expressed mRNAs were also identified, including 1,734 upregulated and 2,350 downregulated ones (Figure 1F, volcano plot). Hierarchical clustering analysis showed that the samples of the model group and the control group could be clearly distinguished, suggesting that the BPD model was successfully constructed and the transcriptome had undergone significant remodeling (Figure 1G, heatmap).

**Figure 1:**
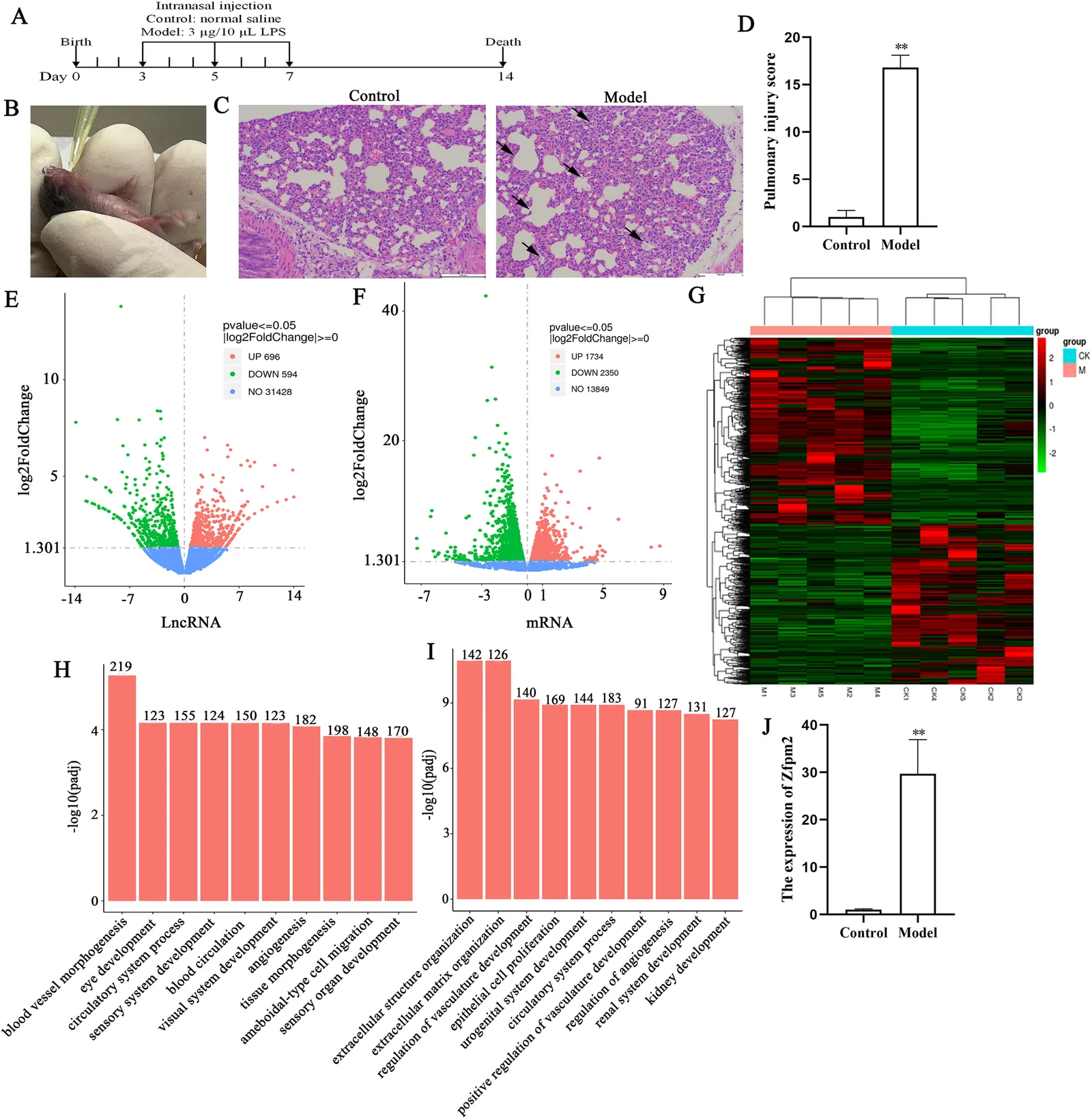
High-throughput sequencing of LncRNA for screening of differentially expressed LncRNAs in the lung tissue of BPD mice **A**: a timeline performed each day up to the day of euthanasia; **B**: Schematic diagram of the construction of the BPD mouse model induced by LPS; **C**: HE staining for observing the pathological changes of lung tissue (scale = 100 μm), the black arrow indicates lung injury; **D**: Lung tissue injury score; **E**: Volcano plot of differentially expressed LncRNAs (|log2FC| > 1, *P* < 0.05); **F**: Volcano plot of differentially expressed mRNAs (|log2FC| > 1, *P* < 0.05); **G**: Clustering heatmap of differentially expressed LncRNAs; **H**: GO functional enrichment analysis of differentially expressed LncRNA target genes; **I**: GO functional aggregation analysis of differentially expressed mRNAs; **J**: qRT-PCR verification of ZFPM2 expression in the lung tissue of BPD mice. **P < 0.01 vs. Control.

To explore the potential functions of the differentially expressed LncRNAs, GO enrichment analysis was performed on the predicted target genes of the differentially expressed mRNAs and LncRNAs. The results showed that the target genes were significantly enriched in lung tissue development and lung epithelial cell proliferation-related items such as blood vessel morphogenesis, tissue morphogenesis, regulation of vasculature development, epithelial cell proliferation, positive regulation of vasculature development, and regulation of angiogenesis (Figures 1H,I). They were also significantly enriched in macrophage chemotaxis and macrophage migration-related biological processes (results not shown). This result suggests that the differentially expressed LncRNAs may regulate macrophage function, thereby affecting the survival of alveolar epithelial cells and lung tissue development.

Given the prominent role of macrophage-related functions in the enrichment analysis， we constructed a LncRNA-mRNA co-expression network to screen upstream LncRNAs regulating macrophage-related genes. Correlation analysis revealed that LncRNA ZFPM2 was significantly negatively correlated with the expression of chemokine-like receptor 1 (Cmklr1) (r = −0.959, *P* < 0.001). Cmklr1 has been confirmed to be a key molecule regulating macrophage polarization and inflammatory chemotaxis (14, 15). Given that Cmklr1 has been proven to be a key molecule regulating macrophage polarization, and that ZFPM2 shows a strong negative correlation with it at the transcriptional level, we hypothesize that Cmklr1 might be a candidate downstream target for the function of ZFPM2. However, this prediction requires further experimental verification. Therefore, we have not included Cmklr1 in the subsequent mechanism studies yet. Instead, we are focusing on the direct effects of ZFPM2 on macrophage polarization and epithelial injury. qRT-PCR verification results showed that the expression of ZFPM2 in BPD mouse lung tissue was significantly increased (Figure 1J, *P* < 0.001 vs. Control), consistent with the sequencing results.

### 3.2 Silencing ZFPM2 induces M2-type polarization of macrophages

Compared with the control group, the expression of ZFPM2 was significantly increased in MH-S cells induced by LPS (Figure 2A, *P* < 0.001). To investigate the effect of ZFPM2 on the function of macrophages, ZFPM2 was silenced in MH-S cells and the regulatory effects on polarization markers and inflammatory factor release were observed. Compared with the control group, three siRNAs could significantly reduce ZFPM2 expression (*P* < 0.001, Figure 2B), among which siRNA1 had the best interference effect and was used for subsequent experiments. As shown in Figure 2C, compared with the control group, the cell viability of the LPS group and the LPS + si-NC group was significantly decreased (*P* < 0.001), while the cell activity was significantly increased after ZFPM2 interference (*P* < 0.001). As shown in Figures 2D–F, compared with the control group, the mRNA and protein expressions of M1-type polarization markers (iNOS, CD86) and the levels of pro-inflammatory factors (TNF-α, IL-1β and IL-6) were significantly increased (all *P* < 0.001), while the mRNA and protein expressions of M2-type polarization markers (CD206, Arg1) and the level of anti-inflammatory factor IL-4 were significantly decreased (all *P* < 0.001), and the level of IL-10 was not significantly changed (*P* = 0.1174). Compared with the LPS + si-NC group, the expression of M1-type markers and pro-inflammatory factor levels in the LPS + si-ZFPM2 group were significantly decreased (*P* < 0.001), while the expression of M2-type markers and anti-inflammatory factors (IL-4, IL-10) were significantly increased (both *P* < 0.001). These results indicate that silencing ZFPM2 can induce the transformation of LPS-stimulated MH-S cells to M2-type.

**Figure 2:**
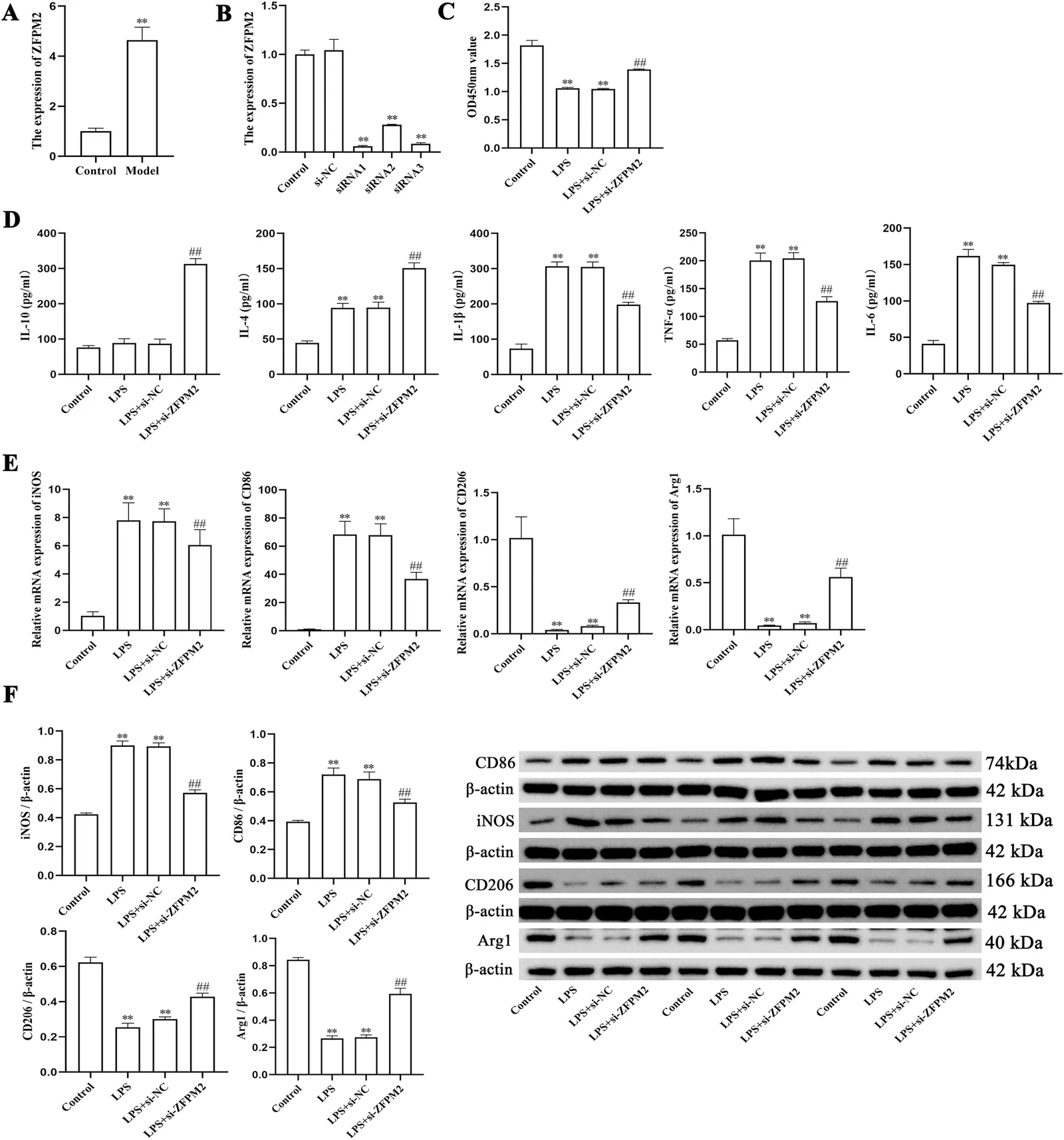
ZFPM2 interference promotes macrophage polarization to M2 type **A**: qRT-PCR verification of ZFPM2 expression level in LPS-induced MH-S cells; **B**: qRT-PCR verification of ZFPM2 interference efficiency in MH-S cells; **C**: CCK8 detection of the activity of each group of MH-S cells; **D**: ELISA detection of the contents of IL-10, IL-4, IL-1β and TNF-α in the supernatant of MH-S cells; **E**: qRT-PCR detection of the mRNA expression of M1 polarization markers (iNOS, CD86) and M2 polarization markers (CD206, Arg1) in MH-S cells; **F**: western-blot detection of the protein expression of M1 and M2 polarization markers. **P < 0.01 vs. Control; ^##^*P* < 0.01 vs. LPS + si-NC.

### 3.3 Silencing ZFPM2 inhibits LPS-induced apoptosis of lung epithelial cells

Further silencing ZFPM2 in MLE-12 lung epithelial cells was conducted to observe its effects on cell viability, apoptosis and inflammasome activity induced by LPS. Compared with the control group, the expression of ZFPM2 was significantly upregulated in the LPS group and the LPS + si-NC group (*P* < 0.001, Figure 3A), cell viability was significantly decreased (*P* < 0.001, Figure 3B), apoptosis rate was significantly increased (*P* < 0.001, Figure 3C), and the protein expressions of caspase-1 and NLRP3 were significantly increased (both *P* < 0.001, Figure 3D), and the levels of IL-1β, IL-6 and TNF-α were significantly increased (all *P* < 0.001, Figure 3E). Compared with the LPS + si-NC group, the cell viability in the LPS + si-ZFPM2 group was significantly increased (*P* = 0.0013), apoptosis rate was significantly decreased (*P* < 0.001), protein expressions of caspase-1 and NLRP3 were significantly decreased (caspase-1: *P* < 0.001; NLRP3: *P* = 0.0456), and the levels of the above inflammatory factors were also significantly decreased (all *P* < 0.001). This result indicates that silencing ZFPM2 can inhibit LPS-induced apoptosis and activation of the inflammasome in lung epithelial cells.

**Figure 3:**
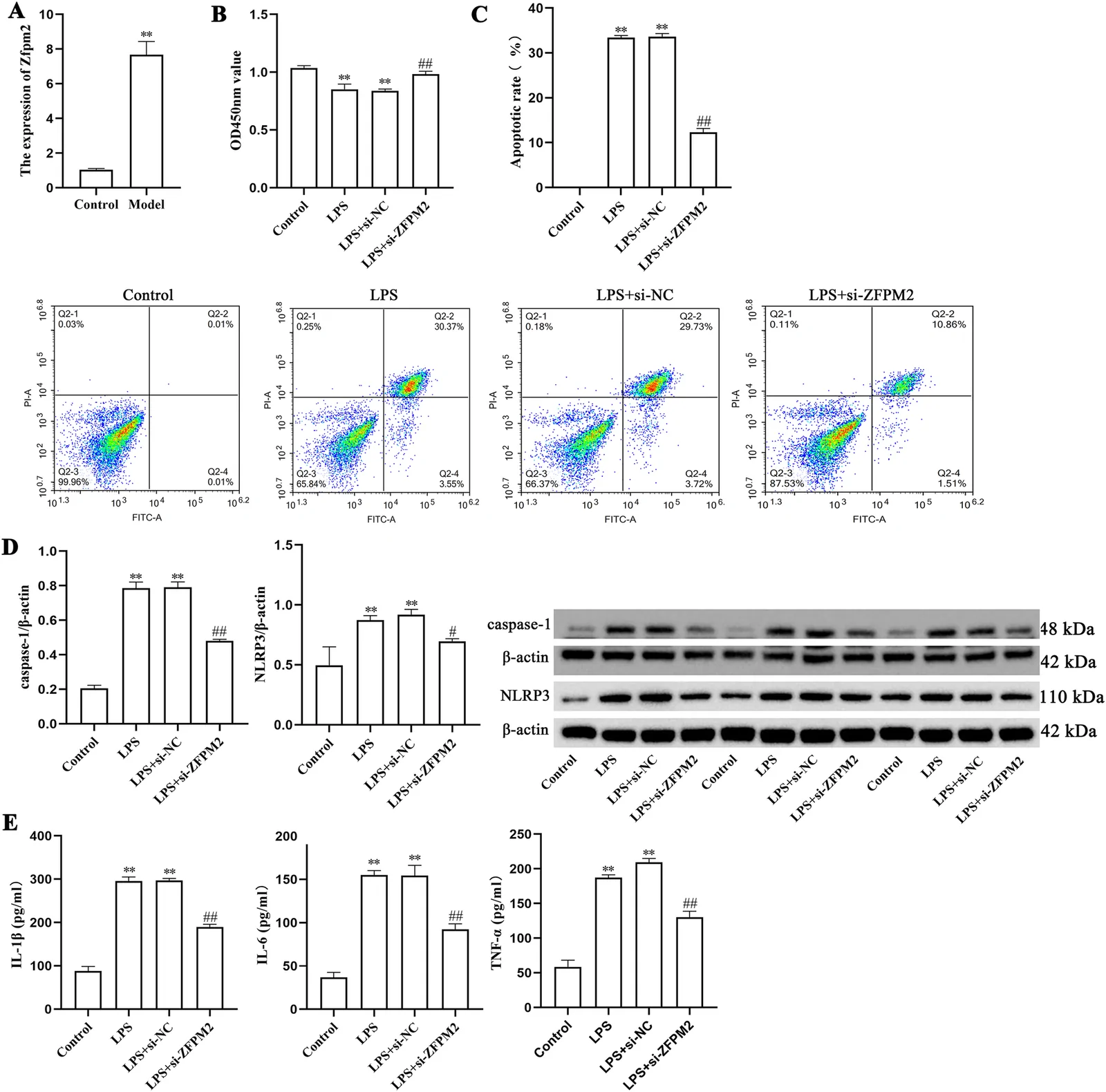
ZFPM2 interference inhibits NLRP3 inflammasome activation and cell apoptosis **A**: qRT-PCR detection of ZFPM2 expression level in LPS-induced MLE-12 cells; **B**: CCK8 detection of the activity of MLE-12 cells; **C**: flow cytometry detection of the apoptosis rate of MLE-12 cells; **D**: western-blot detection of the protein expression of caspase-1 and NLRP3 in MLE-12 cells; **E**: ELISA detection of the contents of IL-1β, IL-6 and TNF-α in the supernatant of MLE-12 cells. **P < 0.01 vs. Control; ^##^*P* < 0.01 vs. LPS + si-NC, ^#^*P* < 0.05 vs. LPS + si-NC.

### 3.4 ZFPM2 silenced macrophages alleviate LPS-induced lung epithelial cell damage

To verify whether ZFPM2 regulates the phenotype of macrophages and thereby affects the fate of lung epithelial cells, a transwell co-culture system of MH-S and MLE-12 was established. The results showed that compared with the control group, the viability of MLE-12 cells significantly decreased (*P* < 0.001, Figure 4A) and the apoptosis rate significantly increased (*P* < 0.001, Figure 4B) after co-culture with MH-S cells in the LPS group and LPS + si-NC group. The expression of caspase-1 and NLRP3 proteins significantly increased (both *P* < 0.001, Figure 4C), and the levels of TNF-α, IL-6, IL-1β, IL-4, and IL-10 significantly increased (all *P* < 0.001, Figure 4D). Compared with the LPS + si-NC group, the viability of MLE-12 cells significantly increased (*P* = 0.0031, Figure 4A) and the apoptosis rate significantly decreased (*P* < 0.001, Figure 4B) after co-culture with MH-S cells in the LPS + si-ZFPM2 group. The expression of caspase-1 and NLRP3 proteins significantly decreased (caspase-1, *P* = 0.0013; NLRP3: *P* = 0.0027, Figure 4C), and the levels of pro-inflammatory factors (TNF-α, IL-6, IL-1β) significantly decreased, while the levels of anti-inflammatory factors (IL-4, IL-10) significantly increased (both *P* < 0.001, Figure 4D). These results indicate that silencing ZFPM2 can regulate the polarization of macrophages to the M2 type, promote the secretion of soluble cytokines, inhibit the apoptosis of lung epithelial cells and the activation of inflammasomes, thereby exerting a protective effect on the epithelial cells.

**Figure 4:**
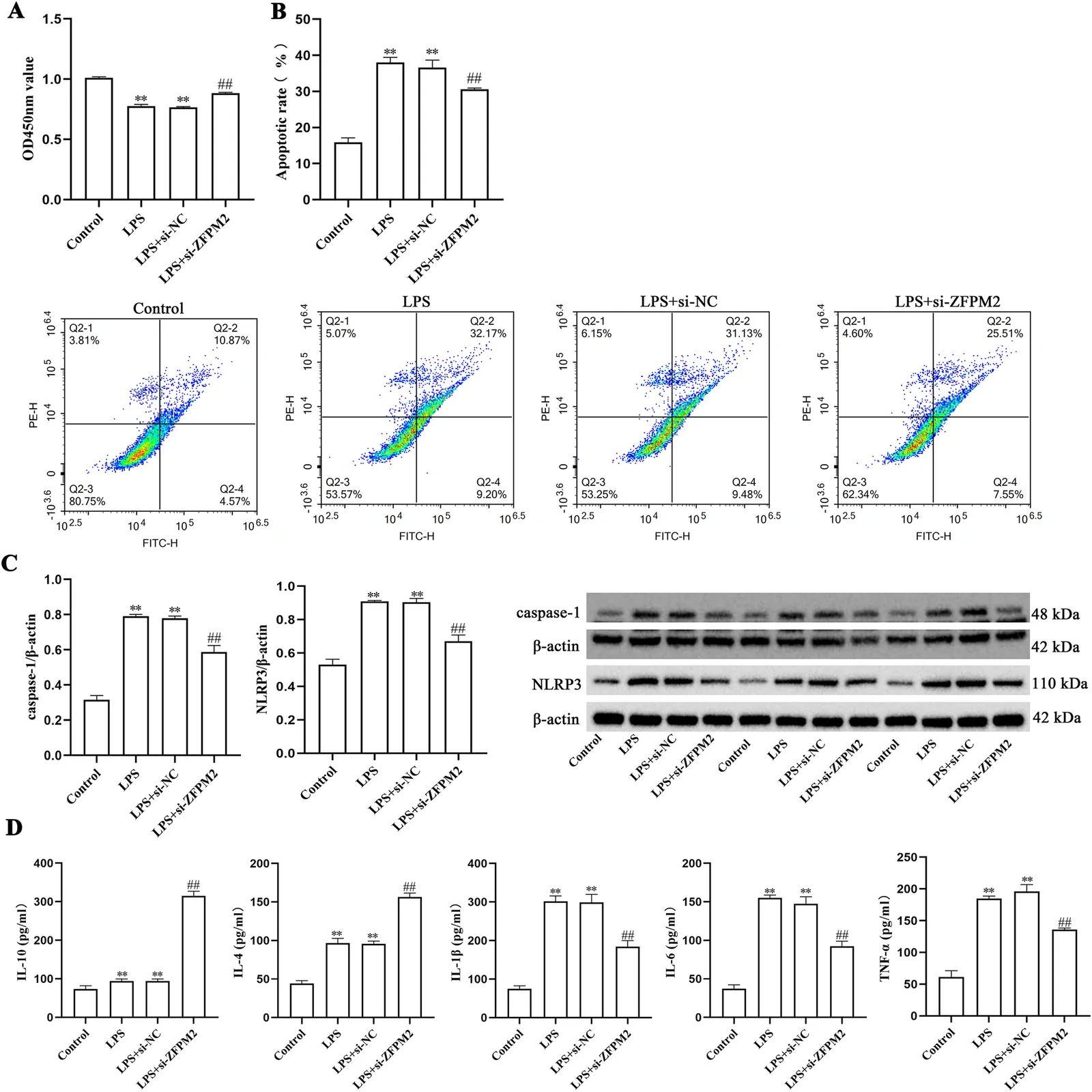
ZFPM2 interference promotes M2 polarization of macrophages and inhibits lung epithelial cell apoptosis **A**: CCK8 detection of the viability of MLE-12 cells in the co-culture system; **B**: flow cytometry detection of the apoptosis rate of MLE-12 cells in the co-culture system; **C**: western-blot detection of the protein expression of caspase-1 and NLRP3 in MLE-12 cells in the co-culture system; **D**: ELISA detection of the contents of IL-10, IL-4, IL-1β and TNF-α in the supernatant of MLE-12 cells. **P < 0.01 vs. Control; ^##^*P* < 0.01 vs. LPS + si-NC.

## 4 Discussion

Bronchopulmonary dysplasia (BPD) is a common chronic inflammatory lung disease in premature infants, which can lead to persistent lung damage, reduce the quality of life of the children, and impose a heavy burden on families and society. The pathological features of the lungs of premature infants with BPD mainly manifest as simplified distal alveoli development, abnormal alveolar formation, vascular proliferation disorder, and pulmonary microvascular remodeling (3). Studies have shown that inflammation is the cores process of BPD occurrence, and IL-1β and TNF-α, as key pro-inflammatory factors, play an important role in this process (16, 17). Lipopolysaccharide (LPS), as a unique component of the cell wall of Gram-negative bacteria, can induce lung inflammation, alveolar simplification, and inhibition of alveolar formation through a single intraperitoneal injection. Its pathological manifestations are similar to those of premature infants with BPD, and thus it is widely used to establish BPD animal models (18). This study based on the LPS-induced BPD mouse model, through lncRNA sequencing, identified 1,290 differentially expressed lncRNAs and 4,084 differentially expressed mRNAs. The functional enrichment analysis of differentially expressed mRNAs and lncRNAs’ target genes showed that these genes not only participate in multiple biological processes such as extracellular structure organization, vascular development, epithelial cell morphology formation and proliferation, vascular morphogenesis and angiogenesis, but are also closely related to macrophage polarization. This suggests that lung epithelial cell development and macrophage polarization may be two key factors in the occurrence and development of BPD, and lncRNAs may affect the pathological process of BPD by regulating these processes.

In recent years, lncRNAs have become a hot topic in the research of various diseases (19). This study through lncRNA sequencing found that LncRNA ZFPM2 was significantly upregulated in BPD lung tissue. Previous studies have reported that ZFPM2 is upregulated in various tumors and participates in regulating tumor cell proliferation, migration, invasion, apoptosis, and epithelial-mesenchymal transition (EMT) (20–22). Chen et al. (23) found that ZFPM2 is closely related to immune cell infiltration in various tumor microenvironments and mainly participates in immune-related pathways in gastric adenocarcinoma, possibly as a prognostic biomarker and immunotherapy target. However, the role of ZFPM2 in BPD has not been reported. This study through qRT-PCR verification found that ZFPM2 was significantly upregulated in BPD mouse model lung tissue and LPS-induced MLE-12 cells. Compared with the LPS + si-NC group, the activity of LPS + si-ZFPM2 group MLE-12 cells was significantly increased, the apoptosis rate was significantly decreased, the expression of caspase-1 and NLRP3 proteins was significantly downregulated, and the levels of IL-1β, IL-6, and TNF-α were significantly decreased. These results indicate that silencing ZFPM2 can alleviate the inflammatory damage related to BPD, but the specific mechanism still needs to be further clarified.

Alveolar macrophages are important immune cells that maintain the homeostasis of lung tissue. The inflammatory response mediated by alveolar macrophages plays a crucial role in the course of BPD. Macrophages can polarize into two subtypes, M1 and M2, under different stimuli, each with pro-inflammatory and anti-inflammatory functions, which are crucial for regulating the inflammatory response and tissue repair in BPD lungs (24). For the infant lungs, macrophages play a dual role in tissue development and immune defense (25, 26). When the lungs are exposed to external stimuli, alveolar macrophages clear endogenous proteins and cell debris through phagocytosis, constituting the first line of immune defense (27). In lung injury models, alveolar macrophages secrete chemokines to attract neutrophils to the lung tissue, triggering an inflammatory cascade and further exacerbating lung injury (28). Multiple studies have shown that the number of innate alveolar macrophages in BPD patients is reduced [29030], and alveolar macrophages show a shift towards a pro-inflammatory phenotype (29). This change disrupts the balance between pro-inflammatory and anti-inflammatory responses, with increased M1-type alveolar macrophages expressing iNOS and secreting pro-inflammatory factors such as IL-1β and TNF-α (30, 31). Alveolar macrophages also participate in supporting vascular development and are the dominant factor in regulating BPD alveolar development arrest, and their reduction may affect the repair process and exacerbate lung injury (32, 33). Therefore, therapeutic strategies targeting macrophages are increasingly attracting attention. Zhang et al. (34) reported that silencing Gm26917 can alleviate LPS-induced inflammatory responses by regulating the function of alveolar macrophages, effectively alleviating acute lung injury. This study found that silencing ZFPM2 can reduce the inflammatory response of macrophages induced by LPS, increase cell activity, and promote macrophage polarization to the M2 type. After co-culturing different treated macrophages with MLE-12 cells, the cell activity of MLE-12 cells increased and the apoptosis rate decreased, suggesting that ZFPM2 may alleviate BPD-related lung injury by mediating macrophage polarization.

The activation of NLRP3 inflammasome has been confirmed as an important step in the occurrence and development of BPD. The activation of the inflammasome can promote the maturation and secretion of pro-inflammatory cytokines such as IL-1β, triggering a strong inflammatory response. Studies have shown that drugs such as dexmedetomidine and dexamethasone can alleviate lung injury induced by inflammation in BPD by inhibiting the activation of NLRP3 inflammasome and the induced pyroptosis (35, 36). The activation of NLRP3 inflammasome is closely related to the function of alveolar macrophages. Zheng et al. (37) found that the Chinese medicine Eclipta prostrata can reduce alveolar macrophage pyroptosis by inhibiting the NLRP3 inflammasome, thereby alleviating alveolar development arrest. Huang et al. (38) reported that the expression and activity of caspase-8 significantly increased in BPD mice. Inhibiting caspase-8 could weaken the activation of NLRP3 inflammasome, reduce the recruitment of CD11b⁺ macrophages and the release of IL-1β and IL-18, and simultaneously alleviate lung epithelial cell apoptosis and improve alveolar development. Consistent with this, our study found that silencing LncRNA ZFPM2 could inhibit the expression of NLRP3 and caspase-1 in LPS-induced MLE-12 cells, and reduce the levels of IL-1β and TNF-α. However, the relationship between the inhibitory effect of interfering ZFPM2 on the NLRP3 inflammasome and the regulation of macrophage polarization is still unclear. Further studies are needed to further clarify the mechanism of ZFPM2 in BPD.

This study has the following limitations: Firstly, this study mainly verified the function of ZFPM2 through *in vitro* cell experiments, lacking animal experiments that specifically interfere with ZFPM2 *in vivo* to further clarify its pathological role in BPD; Secondly, the molecular mechanism by which ZFPM2 regulates macrophage polarization and NLRP3 inflammasome activation has not been fully elucidated. Whether they act independently or have upstream-downstream regulatory relationships needs further exploration; Thirdly, the downstream target genes of ZFPM2 and their interactions with key signaling pathways still need to be further verified through methods such as RNA pull-down and dual luciferase reporter gene experiments; Fourthly, this study did not include clinical samples from BPD patients, and the potential value of ZFPM2 in the clinical diagnosis and prognosis assessment of BPD still needs to be confirmed by subsequent clinical research. Fifthly, this study mainly verified the function of ZFPM2 through a siRNA-mediated knockdown strategy. No overexpression experiments have been conducted to determine whether the upregulation of ZFPM2 is sufficient to drive the pathological phenotypes associated with BPD. In the subsequent research, a ZFPM2 overexpression system will be constructed to further clarify its functional effects.

## 5 Conclusions

In summary, the expression of LncRNA ZFPM2 was significantly upregulated in the lung tissues of BPD mouse models. Silencing ZFPM2 can promote macrophage polarization to the M2 type, inhibit the activation of the inflammasome, alleviate the inflammatory response, and thereby alleviate the lung damage related to BPD.

## Data Availability

Publicly available datasets were analyzed in this study. This data can be found here: DOI: 10.6084/m9.figshare.34057137.
